# PMF-CPI: assessing drug selectivity with a pretrained multi-functional model for compound–protein interactions

**DOI:** 10.1186/s13321-023-00767-z

**Published:** 2023-10-14

**Authors:** Nan Song, Ruihan Dong, Yuqian Pu, Ercheng Wang, Junhai Xu, Fei Guo

**Affiliations:** 1 School of New Media and Communication, Tianjin University, Tianjin, Tianjin, 300072 China; 2 School of Computer Science and Engineering, Central South University, Changsha, 410083 Hunan China; 3 Academy for Advanced Interdisciplinary Studies, Peking University, Beijing, Beijing, 100871 China; 4 College of Intelligence and Computing, Tianjin University, Tianjin, Tianjin, 300350 China; 5 College of Pharmaceutical Sciences, Zhejiang University, Hangzhou, 310058 Zhejiang China; 6 Zhejiang Laboratory, Hangzhou, 311100 Zhejiang China

**Keywords:** Compound–protein interaction, Drug selectivity, Deep learning, Pretrained model

## Abstract

Compound–protein interactions (CPI) play significant roles in drug development. To avoid side effects, it is also crucial to evaluate drug selectivity when binding to different targets. However, most selectivity prediction models are constructed for specific targets with limited data. In this study, we present a pretrained multi-functional model for compound–protein interaction prediction (PMF-CPI) and fine-tune it to assess drug selectivity. This model uses recurrent neural networks to process the protein embedding based on the pretrained language model TAPE, extracts molecular information from a graph encoder, and produces the output from dense layers. PMF-CPI obtained the best performance compared to outstanding approaches on both the binding affinity regression and CPI classification tasks. Meanwhile, we apply the model to analyzing drug selectivity after fine-tuning it on three datasets related to specific targets, including human cytochrome P450s. The study shows that PMF-CPI can accurately predict different drug affinities or opposite interactions toward similar targets, recognizing selective drugs for precise therapeutics.Kindly confirm if corresponding authors affiliations are identified correctly and amend if any.Yes, it is correct.

**Supplementary Information:**

The online version contains supplementary material available at 10.1186/s13321-023-00767-z.

## Introduction

Compound–protein interactions (CPI) play essential roles in biological processes. Since small molecules can modulate the protein’s conformation and affect its functions, it is crucial to find drug compounds binding to protein targets for drug discovery. High-throughput screening in the laboratory is a common approach to finding drug candidates for important targets but it is time-consuming and costing [1].

Previous experimental assays in drug development have accumulated abundant chemoproteomics CPI data. Therefore, researchers devote to developing effective CPI prediction models with advanced deep learning technology [2, 3]. The problem of CPI prediction can be treated as two different tasks, binding affinity regression and binary interaction classification. Affinity regression utilizes validated continuous values such as the half maximal inhibitory concentration ($IC_{50}$). From DeepDTA [4] and DeepAffinity [5] to DeepFusionDTA [6] and MFR-DTA [7], various models take advantage of different deep neural network modules to improve the fitting ability. In spite of quantitative measurement, affinity labels are likely to suffer the data heterogeneous arising from experimental conditions. Classification task considers the interaction relationships between compounds and proteins as simple binary labels. Most of the time the label is divided by an affinity cutoff [8–10]. This task provides a simple and effective way to screen drug candidates, but it is still difficult to define the threshold value for interacting or not, especially for targets with scarce samples. Meanwhile, there are also several models aiming to process both regression and classification tasks with one framework [11–13]. In addition, general CPI models have the potential to apply to more related scenarios.

When screening the lead compounds, one compound is likely to bind to multiple target proteins, especially those with similar active sites. Although multi-target molecules are favorable drug candidates, this target promiscuity may cause unexpected side effects [14]. Therefore, it is essential to focus on drug selectivity when predicting compound–protein interactions.

Conventional works on selective drugs are based on structural analysis. Docking and molecular dynamics simulations help researchers find structural proofs that contribute to selectivity [15]. Although machine learning-assisted methods can process the task of drug selectivity as the classification of molecular properties, they can only predict selective or non-selective drugs toward one target of interest based on available training data. A possible way to integrate bioactivity data from several targets is multi-task learning, which means it can predict drug selectivity toward some targets such as human kinases through a unified model [16, 17]. Another approach is to fit the selectivity window of one drug interacting with two specific targets directly [18]. In that event, there is a bottleneck for predicting the relation of drug selectivity: each aforementioned model has to be constructed on limited interaction data of specified targets, which is hard to generalize to unseen targets.

Typical CPI models follow a Y-shaped architecture, consisting of compound and protein-encoded branches. This indicates that CPI model can detect multiple interactions between different compounds and targets. Recently, the development of CPI framework with generalization ability has raised attention, and pretraining is a desirable technique. There are two ways to introduce pretraining. On the one hand, we can generate embeddings from encoders pretrained on millions of known compounds or protein sequences [19, 20]. Pretrained protein language models such as TAPE [21] and ESM [22] provide a more direct way to transfer associate knowledge for more protein targets and they show capability in CPI prediction [23, 24]. On the other hand, pretraining on large set helps transfer the associated knowledge to specific tasks with the fine-tuning step [25]. Thus, we can exploit these characteristics of pretrained CPI model to detect drug selectivity.

In this paper, we build a novel pretrained multi-functional model for compound–protein interaction prediction (PMF-CPI) and use it for assessing drug selectivity. We introduce the pretrained TAPE module as protein sequence embedding, then take LSTM to process sequences with variant lengths. Meanwhile, we choose GraphSAGE to extract compound features from their topological representations. Kronecker product is used to merge the compound and protein representations. Our model obtained excellent performance on both the regression and classification tasks. In addition, we validated it on CPI activation or inhibition prediction tasks. Finally, we transferred our model to evaluate drugs with target selectivity on three different data sets. Results demonstrate that PMF-CPI has great performance on multiple CPI-related prediction tasks, providing a useful tool for drug discovery.

## Methods

### Data sets

We consider that the problem of compound–protein interaction prediction is related to a wide type of proteins and small molecules, which is not limited to some drugs or protein targets belonging to a specified family. Therefore, we selected two large benchmark datasets with CPI pairs collected from BindingDB database [26].

The first dataset is curated by Karimi et al. [5] for the binding affinity regression task. The pairs are labeled with the negative logarithmic form of the half maximal inhibitory concentration ($pIC_{50}$). The binding affinity $IC_{50}$ values are in molar units. This dataset contains 376,751 compound-protein pairs in total. For the binary classification of CPI, we utilized a benchmark dataset created by Gao et al. [27]. They identified 33,777 positive interactions whose $IC_{50}$ values are less than 100 nM, and 27,493 negative pairs with $IC_{50}>$ 10,000 nM. We followed the same training, validation, and testing splits as creators of the two sets. Besides, Zhang et al. collected a dataset including 11,198 activating or inhibiting drug-target interactions by filtering validated interactions [28]. We also tested the performance of our proposed model on this activation/inhibition mechanism dataset after removing redundancies.

Furthermore, we chose three reported datasets on drug selectivity. The first dataset consists of 1106 compounds with validated activity for $A_1$ and $A_{2A}$ adenosine receptors (AR) [18]. The second one is about four subtypes of Janus kinases (JAK) and we only curated 920 validated inhibitors with $pIC_{50}$ activity values against four JAKs [17]. Third, Xu et al. characterized a series of compounds targeting human cytochrome P450 (CYP). We filtered the compounds with two validated $pIC_{50}$ activity values against CYP3A7 and CYP3A4 for regression, including 3494 interactions. And 7719 interactions with binary labels from curve ranks are used for classification [29].

Collectively, Table 1 presents the numbers of proteins, compounds, and interaction pairs for all the datasets used in the study. The distributions of input lengths, regression values and other statistical descriptions are in Additional file 1: Fig. S1.

**Table 1:** Detailed information of compounds, proteins, and interaction pairs in seven datasets

Datasets	Proteins	Compounds	Label	Pairs	Positive pairs	Negative pairs
BindingDB	2780	265627	$pIC_{50}$	376751	–	–
AR	2	1106	$pEC_{50}$	2212	–	–
JAK	4	920	$pIC_{50}$	3804	–	–
CYP	2	1724	$pIC_{50}$	3494	–	–
BindingDB	813	49752	Interaction	61270	33777	27493
DrugAI	1376	2183	Mechanism	11198	3307	7891
CYP	2	4333	Interaction	7719	4534	3185

### Data representation

Our proposed model takes the sequences of proteins and the simplified molecular-input line-entry system (SMILES) of compounds as primary inputs. First, we transformed the amino acid strings into numerical representations with pretrained module TAPE to vectorize protein sequences. And as for compounds, we converted them to graphs with topological information.

#### Protein sequence

Protein embedding methods are vital for transforming protein sequences into feature-rich representations. In this work, we compared four approaches: one-hot encoding, ProtVec, Tasks Assessing Protein Embeddings (TAPE), and Evolutionary Scale Modeling (ESM).

ProVec, an extension of the Word2Vec algorithm, captures the local sequence information by generating continuous embeddings for fixed-length protein subsequences (also called k-mers) [30]. TAPE is a semi-supervised pretrained framework on protein sequences. This approach allows for the representation of not only individual amino acids but also their context within the protein sequence [21]. ESM is a family of pretrained models that employ the transformer architecture, like BERT, to learn contextual embeddings of protein sequences. These models are trained on large-scale evolutionary data and can capture both local and long-range interactions within the protein [22].

Each of these methods has its advantages and limitations, and the choice of embedding technique depends on the specific problem and the desired level of complexity in the representation. Eventually, we used TAPE to produce 768-dimensionl sequence embeddings.

#### Compound graph

Graph helps in handling the topological structures of compounds. Here, we construct the corresponding molecular map, which clearly reflects the interaction between the internal atoms of the compound [31]. The graph construction and atomic feature extraction process is performed using an open-source cheminformatics software, RDKit (www.rdkit.org). We adopt the atomic feature design of DeepChem [32]. Specifically, the node feature vector is composed of five types of atomic features: atom symbol, atom degree number of bonded neighbors plus the number of hydrogen, the total number of hydrogen, the implicit value of atom, and whether the atom is aromatic. These atomic attributes constitute a multi-dimensional binary feature vector. If there is a bond between them, the edge is set as a pair of atoms. As a result, an indirect binary graph with attribute nodes is constructed for each input SMILES string, which is illustrated in Table 2.

**Table 2:** Node features of molecular atoms

No.	Feature	Dimension
1	One-hot encoding of the atom element	44
2	One-hot encoding of the degree of the atom in the molecule, which is the 11 number of directly-bonded neighbors (atoms)	11
3	One-hot encoding of the total number of H bound to the atom	11
4	One-hot encoding of the number of implicit H bound to the atom	11
5	Whether the atom is aromatic	1
	All	78

### Model architecture

**Fig. 1:**
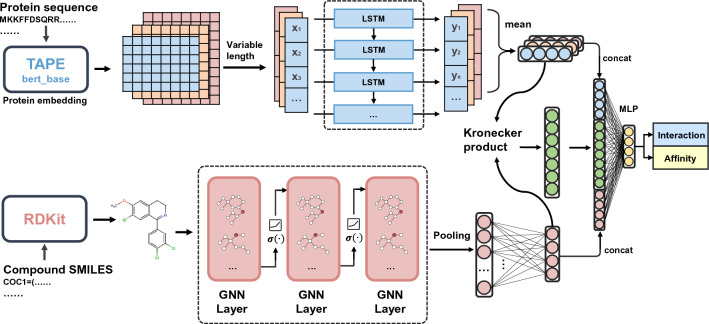
The framework of PMF-CPI. For a compound-protein pair, the protein sequence is transformed into context embedding via pretrained TAPE and then fed into LSTM. And a compound SMILES is turned into a molecular graph by RDKit and encoded with graph neural networks. After that, two encoding vectors are merged through the Kronecker product, and MLP provides a final output. PMF-CPI can process two task types, regression for CPI binding affinities or classification for interactions

Figure 1 shows the framework of PMF-CPI. We carefully choose the encoders of both the protein and compound branches. A variable-length recurrent neural network (RNN) and graph sample and aggregate (GraphSAGE) encoders are adopted for exploiting the features of proteins and compounds, respectively. Then we use the Kronecker product to merge the vectors from two parallel encoders. Finally, the fused vector is fed into two dense layers to produce an output. The output is a continuous value representing $pIC_{50}$ for affinity regression tasks, or a binary value when predicting interactions. Details are as follows.

#### Protein feature encoder

Considering the enriched feature information from embedding the protein sequences, we applied several RNN models to integrate these features in a straightforward manner. Moreover, since the lengths of different sequences vary, we introduced a variable-length RNN module to perform calculations on sequences of different lengths. Given a protein sequence consisting of *L* residues, the node features of the protein graph can be represented by a set $V_p=\{v_i|v_i\in R^h\left( 1\le i\le L\right) \}$, where *h* is the length of the embedding vector $v_i$ produced by the aforementioned embedding methods.

1 $$\begin{aligned} h_p=Mean\sum _{t=1}^{n}{RNNs_{enc}\left( x_t,h_{t-1}\right) } \end{aligned}$$

where $h_p$ is the mean hidden state of protein feature, $h_t$ is the hidden variable output of each input amino acid vector ($x_t$) in the recurrent neural network. We evaluated the performance of different RNNs involving the long-short term memory (LSTM) and gated recurrent unit (GRU), and we chose LSTM at last to extract the deep semantic features of proteins.

#### Compound feature encoder

There are different networks of graph neural networks (GNNs). Graph convolutional network (GCN) can effectively extract features of chemical compounds [33]. Formally, denote a built compound graph as $G=\left( V,E\right)$, where $V\in R^{N\times F}$ is the set of *N* nodes each represented by a *F*-dimensional vector and *E* is the set of edges represented as an adjacency matrix $A\in R^{N\times N}$. A GCN layer is defined as:

2 $$\begin{aligned} H^{{(l+1)}}=\sigma \left( {{\widetilde{D}}}^{-\frac{1}{2}}{\widetilde{A}}{{\widetilde{D}}}^{-\frac{1}{2}}H^{(l)}W^{(l)}\right) \end{aligned}$$

where $H^{(l)}\in R^{N\times F}$ is the output of the previous GCN layer *l*, ${\widetilde{A}}$ is the graph adjacency matrix with added self-loop, ${\widetilde{D}}$ is the graph diagonal degree matrix, and $W^{(l)}\in R^{N\times C}$ is the trainable parameter matrix. Subsequent to the three GCN layers, we added a linear layer to map its output to the same space as the protein branch.

Another graph-based algorithm is GraphSAGE [34]. GraphSAGE generates embeddings for new nodes via sampling and aggregating features learned from a node’s local neighborhood. This algorithm consists of the following steps:

1. Sample a fixed-size neighborhood *S* for each node $v \in V$.
2. Aggregate the features of the sampled neighbors using an aggregation function $AGGREGATE(\cdot )$:
  3 $$\begin{aligned} a_v^{(k)} = AGGREGATE^{(k)}(\{h_u^{(k-1)} | u \in S(v)\}) \end{aligned}$$
  where $h_u^{(k-1)}$ is the feature vector of node *u* at layer $k-1$.
3. Update the node features at layer *k* using the aggregated features:
  4 $$\begin{aligned} h_v^{(k)} = \sigma \left( W^{(k)} \cdot CONCAT(h_v^{(k-1)}, a_v^{(k)})\right) \end{aligned}$$
  where $\sigma (\cdot )$ is an activation function, $W^{(k)}$ is a trainable weight matrix, and $CONCAT(\cdot )$ is a concatenation operation.
4. Repeat steps 2 and 3 for *K* layers to obtain the final node embeddings.

The employment of GraphSAGE facilitates the proficient acquisition and learning of intricate associations among nodes within a specified graph. We adopted the mean aggregator function in our model. The mean aggregator calculates the arithmetic average of the feature values associated with neighbor nodes, thus efficiently encapsulating the local information and retaining the structural information inherent to the graph.

#### Joint module

After encoding two features in the previous section, we constructed a joint representation using the Kronecker product to explicitly capture significant interactions of the compound and protein features.

5 $$\begin{aligned} h_{K}=\left[ \begin{matrix}h_{p}\\ 1\\ \end{matrix}\right] \bigotimes \left[ \begin{matrix}h_{c}\\ 1\\ \end{matrix}\right] \bigotimes \left[ \begin{matrix}h_{c\bigoplus p}\\ 1\\ \end{matrix}\right] \end{aligned}$$

By employing the Kronecker product, we compute the deep binding representations for protein sequences and compounds, and subsequently concatenate the hidden layers of proteins and compounds to form a new vector, denoted as $h_K$. Then a two-layer neural network is utilized to obtain the final output of compound–protein interactions.

6 $$\begin{aligned} Interaction=\sigma \left( \sigma \left( h_kW_1+b\right) W_2+b\right) \end{aligned}$$

This network’s structure is as described in the above formula. To better exploit the subtle signals of compound–protein interactions, we employ LeakyReLU as the activation function $\sigma (\cdot )$.

### Implementation

Our model is implemented in PyTorch [35]. We searched for the best hyperparameters including the batch size in $\{32, 64, 128\}$ and the learning rate in $\{1e-4, 1e-3\}$. And the best combination of batch size and learning rate is 32 and 1e-4. The number of training epochs is 100, and we adopt the early stopping strategy if no improvement within 10 epochs. We set Adam as the optimizer, and dropout rate to be 0.2.

In the compound feature extraction part, we set up three consecutive graph neural network layers. These layers are eventually mapped to 128-dimensional vectors through the mean pooling operation and a linear layer. As for the protein feature encoder, we set the hidden layer dimension of RNNs as 128 as well. Additionally, we utilize different loss functions to accomplish various protein-compound interaction prediction tasks. We adopt the mean squared error (MSE) loss function for regression tasks, while the cross entropy loss function for binary classification.

To evaluate the performance of our proposed model, we take a series of metrics for the regression and classification task. We utilize the MSE, Pearson’s correlation coefficient, and $r_m^2$ to measure the performance of the proposed model. MSE calculates the difference between the predicted values and true labels as follows.

7 $$\begin{aligned} MSE\ =\ \frac{1}{n}\sum _{i=1}^{n}\left( p_i-y_i\right) ^2 \end{aligned}$$

where $p_i$ is the predicted value, and $y_i$ is the corresponding true value.

8 $$\begin{aligned} Pearson=\frac{cov(p,y)}{s(p)s(y)} \end{aligned}$$

In this equation, $cov(\cdot )$ is the covariance between the predicted value *p* and the real value *y*, and $s(\cdot )$ indicates the standard deviation. Another regression metric is $r_m^2$, which have been used in some binding affinity models to evaluate the external predictive performance [4, 7].

9 $$\begin{aligned} r_m^2 = r^2 * (1 - \sqrt{r^2 - r_0^2}) \end{aligned}$$

where $r^2$ and $r_0^2$ are the squared correlation coefficients with and without intercept, respectively.

For classification, the area under the receiver operating characteristic curve (AUC) is the main measurement. Other metrics are the area under the precision-recall curve (AUPR), accuracy, precision, recall, and F1.

## Results and discussion

### Binding affinity regression

We investigated the performance of PMF-CPI under different compound–protein interaction scenarios, and the foremost task is binding affinity prediction. We compared the performance of different modules with the BindingDB regression dataset as the benchmark, then evaluated the predictive capability of our model with other three outstanding models.

#### Evaluation on different modules

**Table 3:** Performance of different modules on the BindingDB regression dataset

Modules	Methods	MSE $\downarrow$	Pearson $\uparrow$	$r^2_m$ $\uparrow$
Protein embedding	one-hot	$0.578\pm 0.003$	$0.856\pm 0.000$	$0.734 \pm 0.002$
	ProtVec	$0.496\pm 0.003$	$0.879\pm 0.000$	$0.773 \pm 0.001$
	**TAPE**	${\varvec{0.474\pm 0.003}}$	$0.884\pm 0.001$	$0.782 \pm 0.001$
	ESM	${\varvec{0.474\pm 0.003}}$	${\varvec{0.885\pm 0.001}}$	${\varvec{0.783 \pm 0.001}}$
Feature encoder	GCN-RNN	$0.485\pm 0.002$	$0.882\pm 0.001$	$0.778 \pm 0.001$
	GCN-GRU	$0.490\pm 0.003$	$0.880 \pm 0.001$	$0.775 \pm 0.001$
	GCN-LSTM	$0.485\pm 0.004$	$0.883\pm 0.001$	$0.780 \pm 0.002$
	GraphSAGE-RNN	$0.477\pm 0.003$	$0.884\pm 0.001$	$0.782 \pm 0.002$
	GraphSAGE-GRU	$0.477\pm 0.004$	$0.884\pm 0.001$	$0.782 \pm 0.002$
	**GraphSAGE-LSTM**	${\varvec{0.474\pm 0.003}}$	${\varvec{0.884\pm 0.001}}$	${\varvec{0.782 \pm 0.001}}$
Joint module	Concatenate	$0.570\pm 0.004$	$0.859\pm 0.001$	$0.738 \pm 0.002$
	**Kronecker product**	${\varvec{0.474\pm 0.003}}$	${\varvec{0.884\pm 0.001}}$	${\varvec{0.782 \pm 0.001}}$

We conducted several comparative experiments to compare the performance of various modules, including the embedding methods of protein sequence, feature encoders, and joint module strategies. The results are listed in Table 3, where the standard errors are estimated by bootstrapping over 10 times. Our final used modules and best metric values are marked in bold.

We compared four different protein sequence embedding methods, which have been introduced in the section Data representation. Among them, the simplest one-hot sequence embedding shows the highest MSE of 0.578 and ProtVec performs better. Two pretrained language models TAPE and ESM contribute to improved MSE and Pearson compared with one-hot and ProtVec. Based on large amounts of sequences, pretrained models have learned abundant associations in protein repertoires that are useful for embeddings. Although the model with ESM performed slightly better than which with TAPE for 0.001 improvements on the Pearson, we decided to use TAPE for the final architecture of PMF-CPI. This is because TAPE produced a lighter representation (768-dimension) than ESM (1280-dimension), which means less storage and higher computing speed.

As for encoders, we tested the combinations of two GNNs for compounds (GCN and GraphSAGE) and three variants of RNNs for protein embeddings. Table 3 shows the regression results of these six architectures. GraphSAGE is more effective in extracting features from chemical compounds in comparison to GCN, with lower MSE and higher Pearson. Three RNN variants do not show significant differences, while LSTM is slightly better than the naive RNN and GRU; thus, we took the GraphSAGE and LSTM as final encoders of PMF-CPI. Most CPI models use simple concatenation to fuse compounds and protein embedding vectors. Here we selected the Kronecker product as the joint module to enrich interaction information between a compound-protein pair. The comparison results of these two fusion approaches suggest that the Kronecker product can improve the fitting results greatly. For instance, the MSE value improves from 0.570 to 0.474.

#### Comparison with other methods

To benchmark the performance of our proposed method against existing approaches, we assessed our model on the BindingDB regression dataset and compared the results with representative models DeepAffinity, DeepDTA, and MONN. DeepAffinity is a unified RNN-CNN model to predict compound-protein affinity, and DeepDTA utilizes two CNN encoders for compound SMILES and protein sequences. MONN extracts the non-covalent interactions from available complex structures. With pretrained sequence context embeddings and the graph neural network, our proposed model obtained an MSE of 0.474 and Pearson correlation coefficient 0.884. Table 4 shows the results in comparison to the aforementioned methods, and PMF-CPI demonstrates a significant improvement.

In addition, we employed an ensemble technique by integrating diverse sets of parameters identified during the training stage. The results are also recorded in Table 4. Following the ensemble strategy of MONN, the “parameter ensemble” is the average result of the last ten epochs, and “NN ensemble” means the average of three networks with different hyperparameters [11]. We found that the ensemble approach led to a better predictive performance on the BindingDB regression dataset. And our model still performs best among these approaches, with an MSE of 0.416 and a Pearson of 0.899. This indicates that PMF-CPI has an excellent performance in predicting compound-protein binding affinities.

**Table 4:** Regression performance of PMF-CPI in comparison with three representative methods

Methods	MSE $\downarrow$	Pearson $\uparrow$
DeepAffinity	0.548	0.840
DeepDTA	0.612	0.848
MONN	0.584	0.858
PMF-CPI	**0**.**474**	**0**.**884**
DeepAffinity(Parameter Ensemble)	0.533	0.840
DeepAffinity(NN Ensemble)	0.504	0.860
DeepDTA(Ensemble of 30 Models)	0.471	0.886
MONN(Ensemble of 30 Models)	0.432	0.895
PMF-CPI(Parameter Ensemble)	**0**.**416**	**0**.**899**

### Compound–protein interaction classification

We set two different classification tasks for compound–protein interactions. First, we trained and evaluated our proposed model on conventional CPI prediction, which means to predict whether the molecule pairs interact or not. Then we tested it on the DrugAI dataset which categorizes compound–protein interactions into activation or inhibition mechanisms. PMF-CPI obtained good performance on these two classification tasks.

#### Interacting/non-interacting prediction

**Fig. 2:**
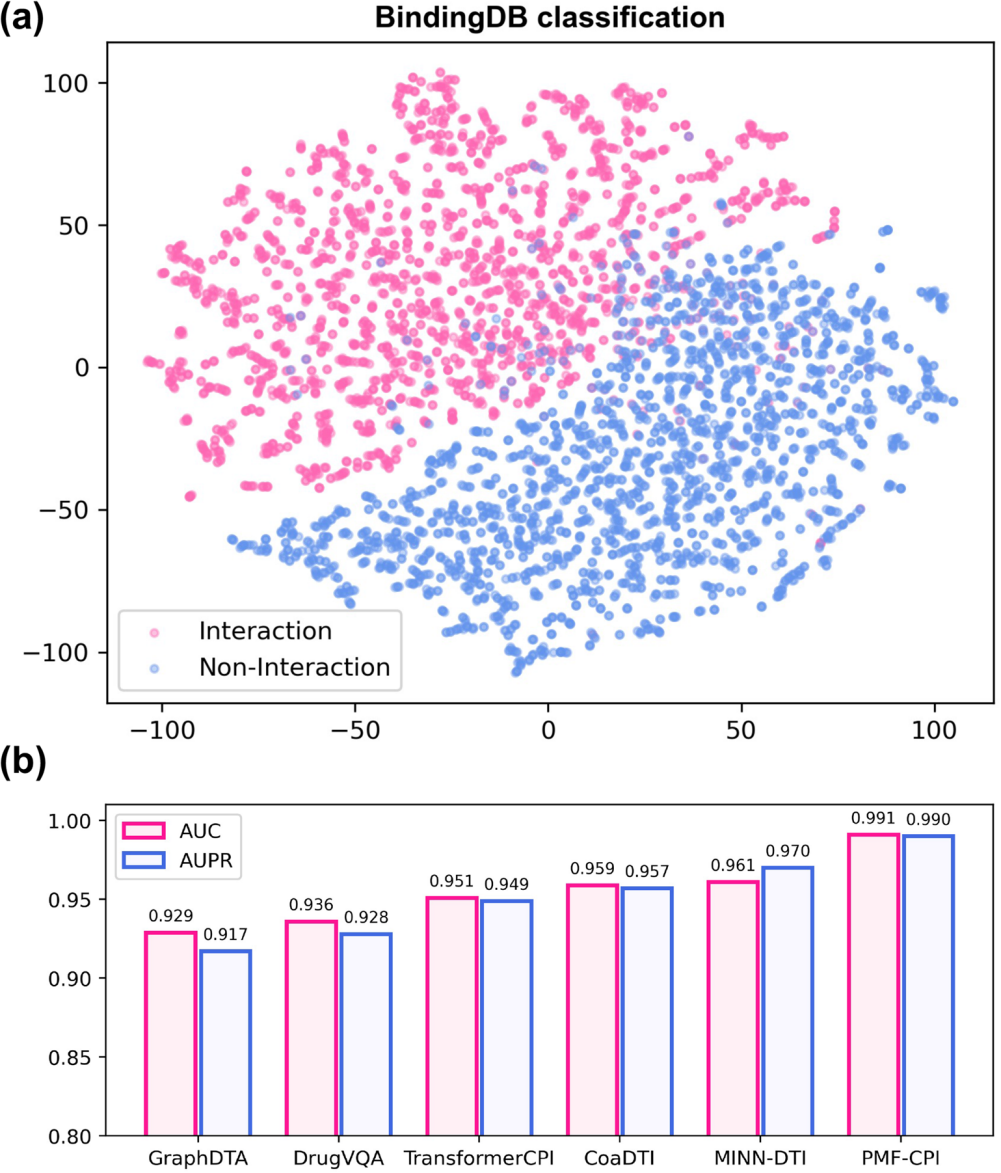
Results of CPI Classification. **a** Visualization of the representation via t-SNE. **b** Comparison with other methods. PMF-CPI has the highest AUC and AUPR values among six models

Using the parameters that contributed to the best performance in the regression experiments, our model also showed reliable performance on the BindingDB classification dataset, achieving an AUC of 0.991. Besides, our model demonstrated strong performance on other metrics. The AUPR reaches 0.990, the accuracy is 0.966, the precision is 0.965, and the recall is 0.968. We represent the two-dimensional feature space by t-distributed stochastic neighbor embedding (t-SNE) [36] in Fig. 2a. Our model can effectively distinguish the active interactions and the inactive samples.

Figure 2b shows the results of PMF-CPI in comparison with other state-of-the-art CPI models. Our AUC value of 0.991 is significantly higher than which of other models. Notably, DrugVQA and MINN-DTI have used the protein distance map, which is the simplified representation of protein structures. And the rest baselines designed different attention-based layers to capture the connections between molecules [37]. However, PMF-CPI is an attention-free framework without adding protein structural information, and it still reached such an AUC value. This indicates the contribution of pretrained protein language model TAPE in the part of feature characterization.

**Fig. 3:**
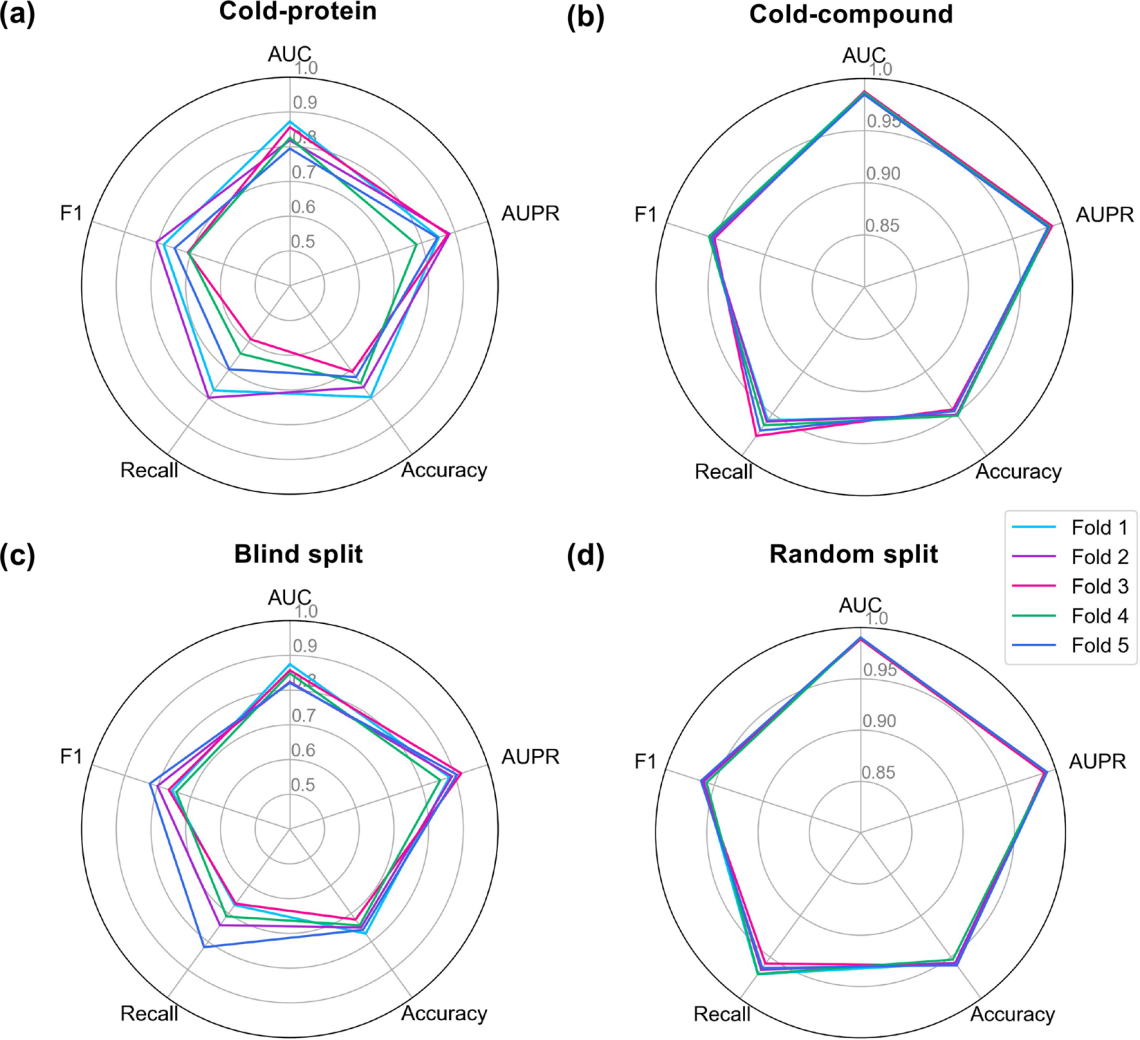
Five-fold cross-validation of PMF-CPI on BindingDB classification dataset. **a** Cold-protein splitting. **b** Cold-compound splitting. **c** Blind splitting, i.e., both the protein and compound that appear in the test set are not seen by the model at the training stage. **d** Random splitting, i.e., split by compound–protein interactions randomly

Meanwhile, we tested the performance of PMF-CPI under four different split settings to evaluate the generalization of the model. The cold-protein setting means dividing the interactions into training and test sets according to their proteins, for proteins that appear in the test set are absent in the training set. Likewise, every compound in the test set of the cold-compound setting is absent from the training set. For blind splitting, both the compounds and proteins in the test set are unseen by the model during the training stage [19, 38]. Fig. 3 shows the five-fold cross-validation results on the BindingDB classification dataset. The settings of cold-protein, cold-compound, and blind split are more challenging scenarios for the CPI model, and their performances decrease in varying degrees. Since the number of compounds in used datasets is far larger than that of proteins, the performance of cold-protein split declined drastically. This trend also appeared in the regression task (Additional file 1: Table S1). To some extent, cold-protein and blind split displayed fluctuations on five folds (Fig. 3a and c), while cold-compound and random split obtained high and stable metrics, including mean AUCs of 0.986 and 0.990, respectively (Fig. 3b,  d, and Additional file 1: Table S2). PMF-CPI still got an AUC of 0.845 and an AUPR of 0.891 on average under the blind split, indicating it can be applied to the prediction of entirely new interactions.

#### Activating/inhibiting prediction

Most CPI prediction works focus on the binding relationship between compounds and proteins. Specifically, the modulation role of drugs play on protein can be divided into activation and inhibition. Based on the reported dataset named DrugAI, we evaluated our model’s performance using a five-fold cross-validation strategy to validate the predictive capability.

**Fig. 4:**
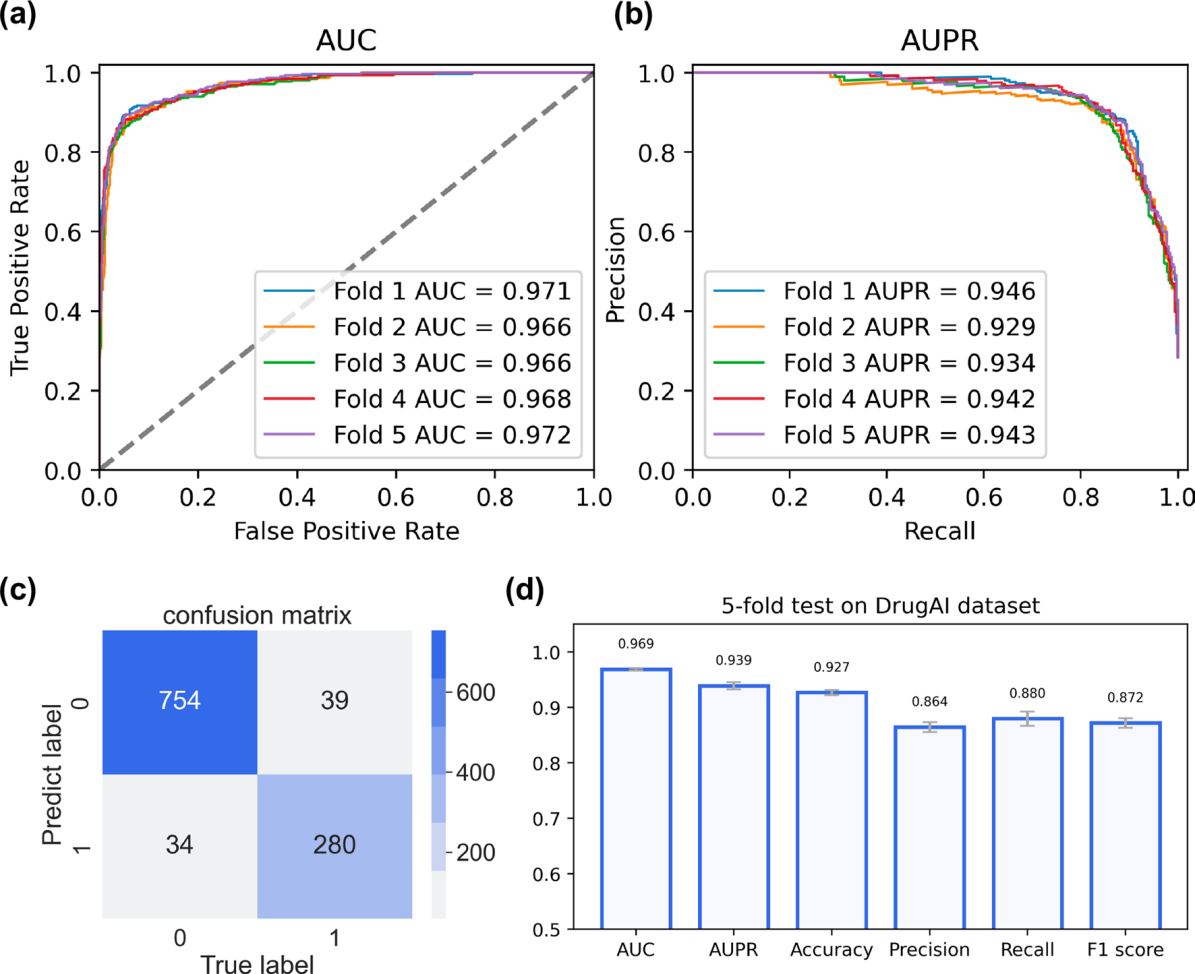
The AUC and AUPR of five-fold cross-validation on DrugAI dataset. The highest fold has an AUC of 0.972 and an AUPR of 0.946, which suggests that PMF-CPI can classify activating or inhibiting interactions accurately

Figure 4a and b shows the classification results over five folds. Our model achieved the highest AUC of 0.972 and an AUPR of 0.946. Compared with the AUC value 0.971 of the multi-view deep learning model DrugAI, our plain architecture can yield a competitive performance on this mechanism task. Figure 4c displays the confusion matrix of PMF-CPI on the test set, annotating the number of true positives 280, true negatives 754, false positives 34, and false negatives 39. Our model reached an accuracy of 0.927 and a precision of 0.864 (Fig. 4d). We also used the cold-protein, cold-compound, and blind split methods for different five-fold splittings on this dataset and the results are recorded in Additional file 1: Table S3. Similar tendencies emerged here for which new proteins had bigger impacts than new compounds. The mean AUC of the blind split was 0.846 when predicting the regulatory mechanisms in compound–protein interactions. In general, PMF-CPI has the potential to solve multiple CPI-related tasks.

### Analysis on drug selectivity

The above results have demonstrated that our model obtained outstanding performance on BindingDB regression and classification benchmarks. In this section, we will explore predicting drug selectivity after fine-tuning the model on specific datasets.

#### Fine-tuning on selective data

Here, the datasets are from three different backgrounds of drug selectivity. Expressing in the brain regions, adenosine receptors (AR) are important targets of neurodegenerative diseases. Two subtypes $A_1$ and $A_{2A}$ have opposite effects on cAMP levels; thus, their selective antagonists play different regulatory roles [39]. Tyrosine kinase JAKs are crucial parts of the JAK/STAT signaling pathway that regulates inflammatory responses, cell apoptosis, etc. Sharing high sequence homology, four members of JAKs may be induced via the same compounds, which leads to unsafe side-effects [40]. Human cytochrome P450s (CYP) are important protein targets related to metabolism. In the subfamily 3A, CYP3A7 and CYP3A4 are liver enzymes that exist in infants and adults, respectively [41]. Hence, predicting selective drugs of them is meaningful to precise therapeutics.

**Fig. 5:**
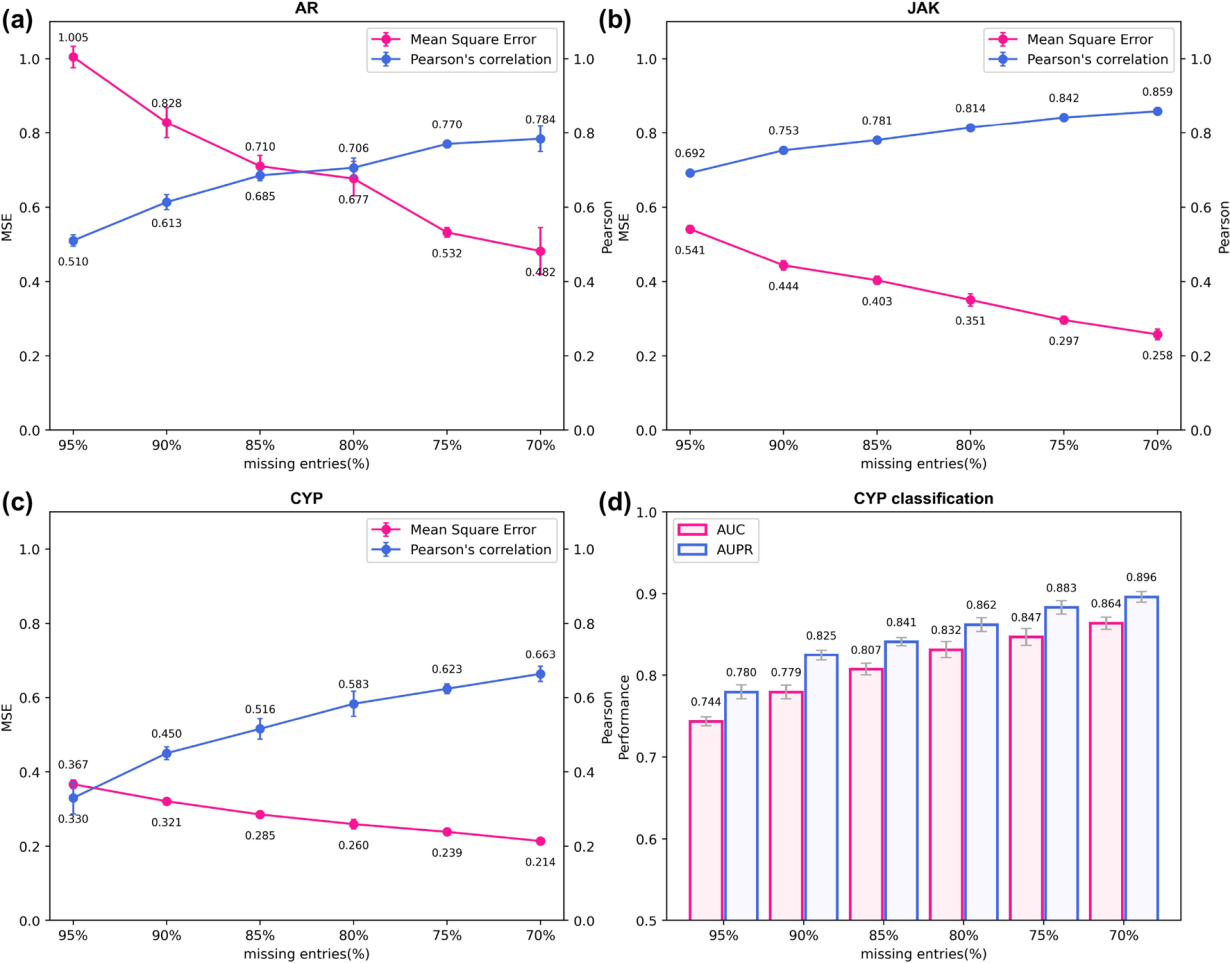
Fine-tuning results of PMF-CPI on datasets about drug selectivity. Different fine-tuning set scales are used with missing entries from 70% to 95%. MSE and Pearson’s correlation coefficient are shown for regression sets **a** AR, **b** JAK, and **c** CYP. **d** AUC and AUPR are used for CYP classification. All results are from five-fold cross-validation with the standard deviation as error bars

Compared with training a model for selectivity prediction from scratch, fine-tuning saves time and can reach a good predictive performance with limited training data. We conducted a series of experiments with different fractions of missing data on four datasets, three for regression (Fig. 5a–c) and one for classification (Fig. 5d). When the ratio of missing entries reduces, the predictive performances improve. AR got an MSE of 1.005 when only training on 5% activity data. If the seen ratio improves to 30%, its MSE value drops more than 0.5 and Pearson increases to 0.784. Since JAK and CYP datasets contain more samples over 3000, they have lower MSEs with 95% missing entries. For regression, our model can reach MSEs of about 0.2 and Pearson’s correlation coefficient of around 0.8 when only training on 30% of each dataset. For classification, PMF-CPI yields an AUC of 0.864 and an AUPR of 0.896. By contrast, we found it unsatisfactory to predict these selectivity sets using the pretrained model solely or separating out a fraction of samples for direct training (Additional file 1: Table S4 and Fig. S2). This suggests that our pretrained model with fine-tuning can provide an accurate and direct prediction of the binding affinity and interaction relationship for selective drugs toward similar targets.

#### Case study of CYP3A4/CYP3A7 inhibitors

Xu et al. assayed almost 5000 drugs and drug-like compounds toward CYP3A4 and CYP3A7 via quantitative high-throughput screening and identified multi-target or selective inhibitors. Based on these data, they constructed machine learning models to detect CYP3A4 or CYP3A7 selective inhibitors [29]. Without utilizing target information, they have to build models for each target and do not develop a useful regression model with activity data. Since our CPI model receives both the molecule and protein inputs, it can produce the affinity or interaction of one drug toward two targets at the same time, and the outputs can be analyzed for selectivity.

**Fig. 6:**
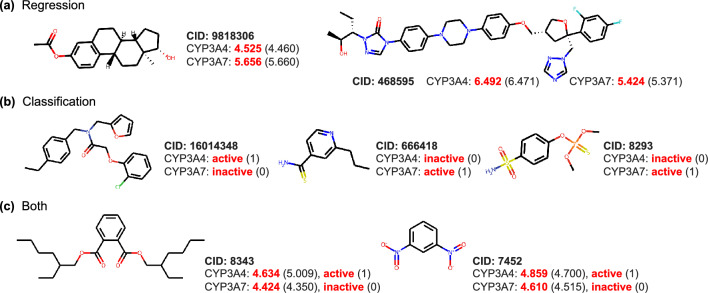
Representative CYP3A4/CYP3A7 selective inhibitors from test sets. PubChem CID are marked with molecular structures. Predictions against two targets are in red, while true labels are in the brackets

We fine-tuned our model on the CYP-related sets. Results in Fig. 5c and d demonstrate that our model can effectively predict the affinity or inhibitory relationship with only seeing less than 30% samples. Belonging to the human CYP3A subfamily, CYP3A4 and CYP3A7 share the sequence identity of 88.5% and similarity of 94.4% (alignment shown in Additional file 1: Fig. S3). But some compounds exhibit distinct inhibitory affinity against these two similar targets, for example, two molecules shown in Fig. 6a. The validated $pIC_{50}$ values of molecule 9818306 are 4.460 and 5.660 for CYP3A4 and CYP3A7, respectively, which means their difference in activity is more than an order of magnitude. For such compounds, our model can predict the affinity accurately with an error of less than 0.1.

There are also some interaction pairs without detailed $pIC_{50}$ values in CYP sets that suit for the classification model. Some molecules are labeled oppositely toward two targets, which indicates that they are specific drugs for infants or adults. Figure 6b displays three cases. Molecule 16014348 is active for only CYP3A4 while 666418 and 8293 are active for CYP3A7. The predictions from our fine-tuned classification model are consistent with the actual labels.

More importantly, the prediction of precise affinity values and binary interaction relationships can compensate for each other. We list two compounds in Fig. 6c. Molecule 8343 and 7452 own similar activity when binding to CYP3A4 or CYP3A7. If we distinguish their inhibitory activity with a specific threshold such as five, we may not identify two molecules as selective inhibitors. Nevertheless, their active labels are comprehensive classifications according to not only the activity but also the efficacy and curve-fitting confidence. Hence, the processed data mark two molecules as CYP3A4 selective inhibitors. When inputting our model, both the predictions from regression and classification models help to recognize the proper selective relations.

## Conclusions

In this study, we proposed a compound–protein interaction prediction model called PMF-CPI by leveraging the protein language model embedding and molecular graphs. The main contributions of PMF-CPI are as follows: (1) PMF-CPI utilizes the embedding of a large language model of proteins, which not only fully captures the protein features but also reduces the requirement for computational resources; (2) PMF-CPI is a multi-functional framework for CPI prediction. This model achieves promising performance on binding affinity regression compared with other methods. Meanwhile, it can be used in classification tasks including recognizing active interaction pairs and distinguishing activation or inhibition mechanisms; (3) PMF-CPI provides a pretrained model for assessing drug selectivity. After being fine-tuned on selective datasets related to special targets, PMF-CPI can accurately predict different drug interactions or affinity values. In short, PMF-CPI suits for few-shot learning, instead of case-by-case construction on targets of interest.

There are also some limitations of our proposed model. First, we just explored the graph representations of compounds, instead of integrating different encodings from multiple levels such as strings and molecular fingerprints. As an attention-free model, PMF-CPI has reached a prominent prediction accuracy consisting of the GraphSAGE and LSTM modules, but its interpretability still needs improvement without the aid of attention layers. Second, we trained PMF-CPI for regression or classification on different datasets separately and it cannot give the evaluation of compound-protein affinity and interaction labels in one run. Finally, although PMF-CPI provides a unified pretrained model for target-specific selectivity tasks, fine-tuning is a crucial step to obtain better performance. From this point of view, domain adaptation, multi-task learning, and other techniques are worth exploring for data heterogeneous and scarcity. We explore the possibility of the CPI framework on the issue of drug selectivity in this work, and we hope that CPI models can harness the strengths in more real-world scenarios of drug discovery.

### Supplementary Information

**Additional file 1.** Additional figures and Tables.

## Data Availability

All the codes and data used in this article can be found in this github repository: https://github.com/guofei-tju/PMF-CPI.

## Abbreviations

AR: Adenosine receptors
AUC: Area under the receiver operating characteristic curve
AUPR: Area under the precision-recall curve
BERT: Bidirectional encoder representation from transformers
CPI: Compound–protein interaction
CYP: Cytochrome P450
ESM: Evolutionary scale modeling
GRU: Gated recurrent unit
GCN: Graph convolutional network
GNN: Graph neural network
GraphSAGE: Graph sample and aggregate
$IC_{50}$: Half maximal inhibitory concentration
JAK: Janus kinases
LeakyReLU: Leaky rectified linear unit
LSTM: Long-short term memory
MSE: Mean squared error
RNN: Recurrent neural network
SMILES: Simplified molecular-input line-entry system
TAPE: Task assessing protein embeddings
t-SNE: T-distributed stochastic neighbor embedding
