# Supplementary material for: PMF-CPI: assessing drug selectivity with a pretrained multi-functional model for compound–protein interactions
Source: J Cheminform. 2023 Oct 14;15:97. doi: 10.1186/s13321-023-00767-z (PMC10576287; doi:10.1186/s13321-023-00767-z)
Supplement: Supplementary file 1 — Additional file 1. Additional figures and Tables. [file 13321_2023_767_MOESM1_ESM.pdf]

Additional file 1

PMF-CPI: Assessing drug selectivity with a pretrained multi-functional model for compound-protein interactions

Nan Song[1,4†], Ruihan Dong[3†], Yuqian Pu[4], Ercheng Wang[5,6*], Junhai Xu[1,4*], Fei Guo[2*]

[1] School of New Media and Communication, Tianjin University, Tianjin, 300072, Tianjin, China.

[2] School of Computer Science and Engineering, Central South University, Changsha, 410083, Hunan, China.

[3] Academy for Advanced Interdisciplinary Studies, Peking University, Beijing, 100871, Beijing, China.

[4] College of Intelligence and Computing, Tianjin University, Tianjin, 300350, Tianjin, China.

[5] College of Pharmaceutical Sciences, Zhejiang University, Hangzhou, 310058, Zhejiang, China.

[6] Zhejiang Laboratory, Hangzhou, 311100, Zhejiang, China.

[*] Corresponding authors. E-mail: wangec@zju.edu.cn; jhxu@tju.edu.cn; guofei@csu.edu.cn

[†] These authors contributed equally to this work.

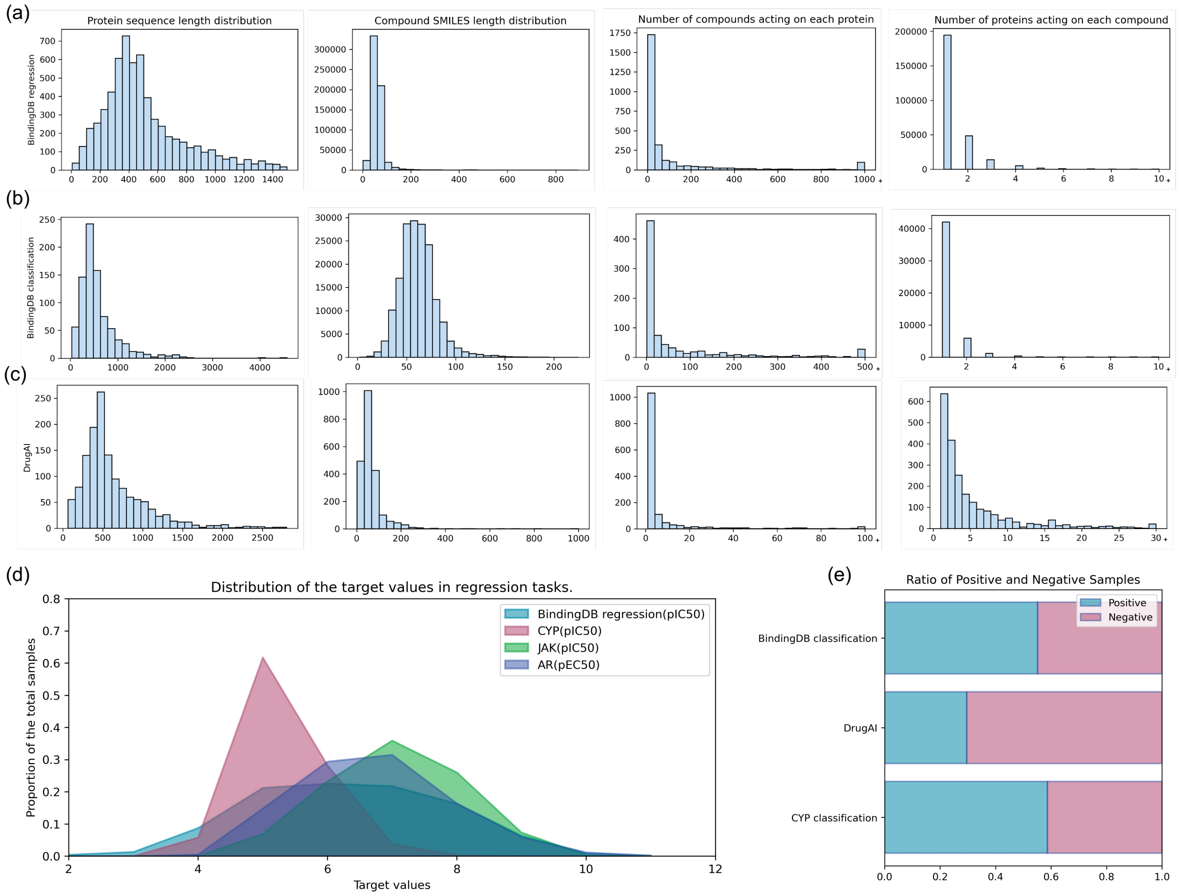

Fig S1. The statistical description of datasets. Four columns show the distribution of protein sequence lengths, compound SMILES lengths, the number of compounds interacting with each protein, the number of proteins interacting with each compound, respectively. Three general datasets shown here are (a) BindingDB regression set; (b) BindingDB classification set; (c) DrugAI activating/inhibiting set. (d) Label distribution for regression datasets, including BindingDB regression and three selectivity-related sets. (e) Positive and negative labels for classification sets involving the BindingDB classification set, DrugAI set, and CYP classification set.

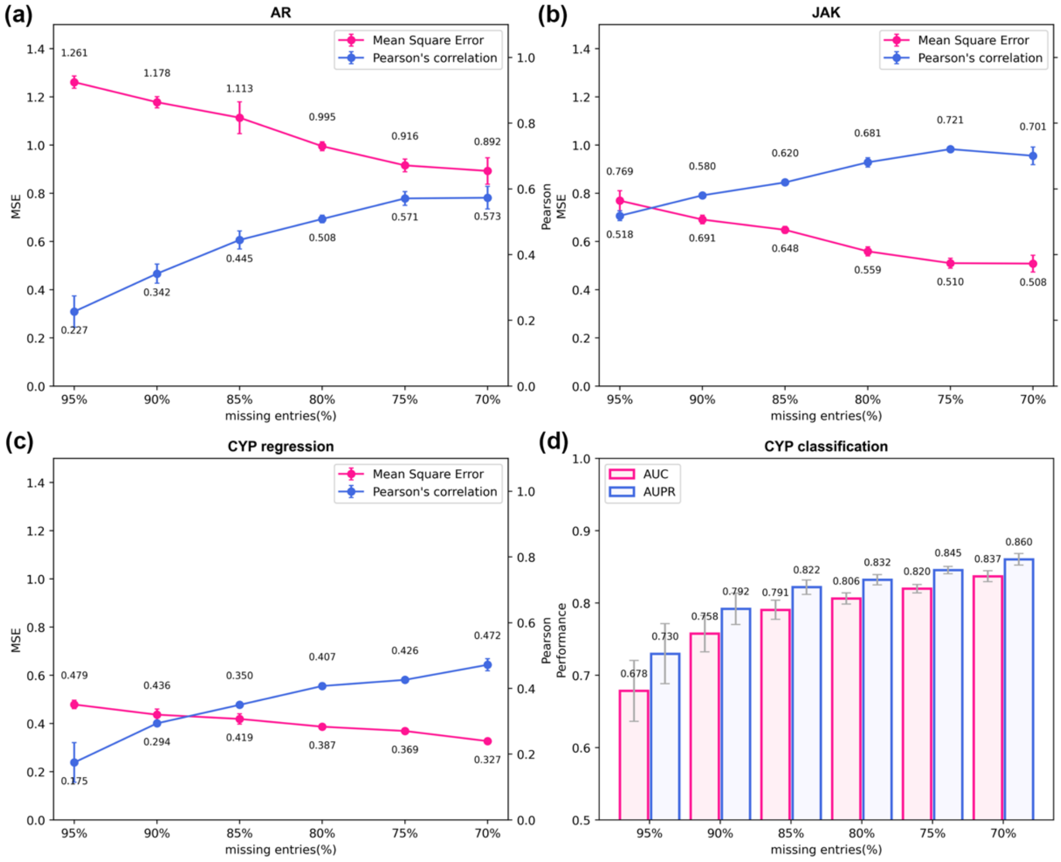

Fig S2. Results of PMF-CPI trained on four datasets about drug selectivity directly. MSE and Pearson's correlation coefficient are shown for regression tasks in (a) AR, (b) JAK, and (c) CYP. (d) AUC and AUPR are used for CYP classification. Experiments follow the same settings in Fig. 5 but are not initialized from pretrained weights. All results are from five-fold cross-validation with the standard deviation as error bars.

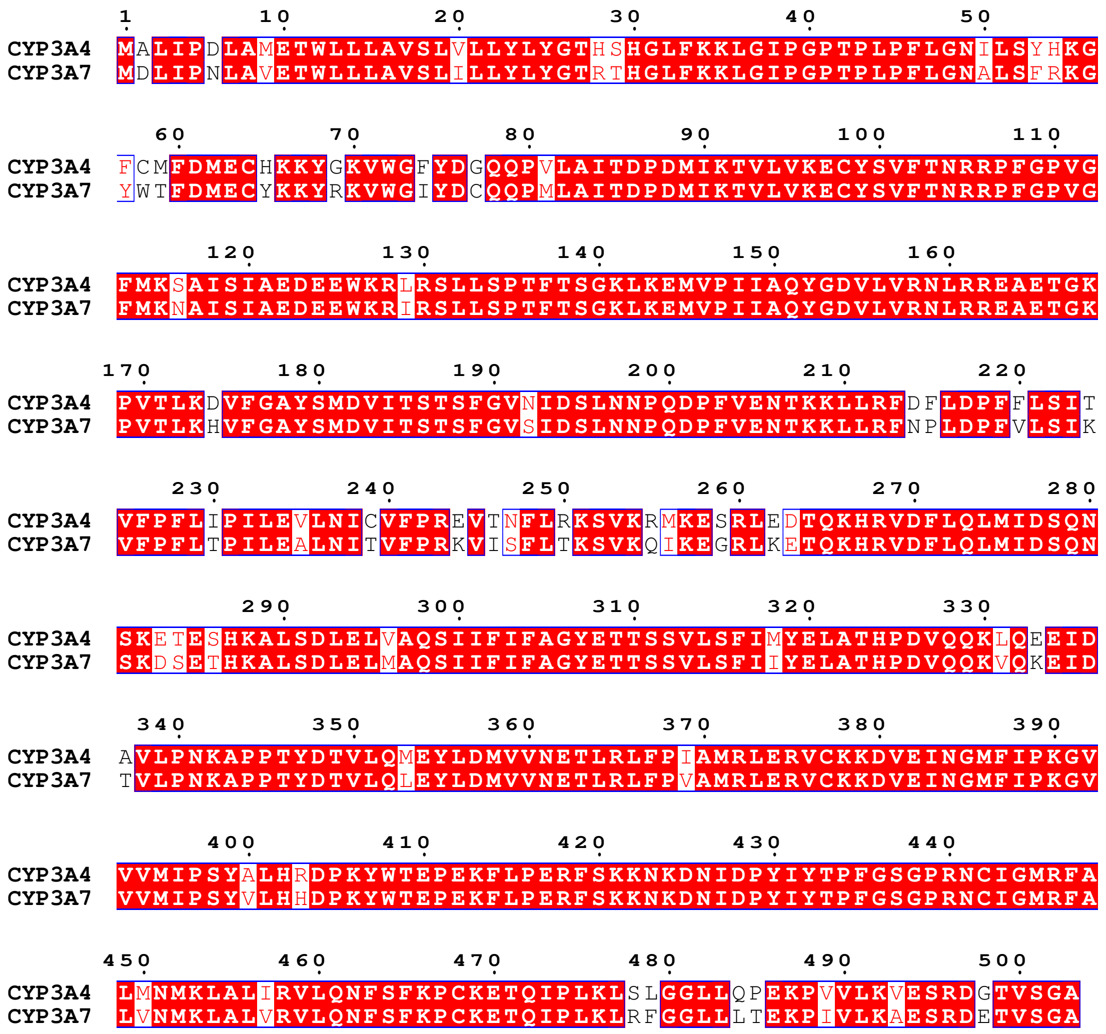

Fig S3. Sequence alignment of human CYP3A4 (P08684) and CYP3A7 (P24462). The alignment is processed with the Needleman-Wunsch algorithm and BLOSUM62 scoring matrix. Visualization by ESPript3.0.

Table S1. Five-fold results of PMF-CPI on BindingDB regression dataset under different splitting methods.

Split methods	MSE (std)	Pearson (std)
Cold-protein	1.566 (0.110)	0.541 (0.024)
Cold-compound	0.492 (0.007)	0.880 (0.002)
Blind split	1.689 (0.124)	0.500 (0.031)
Random split	0.483 (0.003)	0.883 (0.001)

Table S2. Five-fold results of PMF-CPI on BindingDB classification dataset under different splitting methods.

Split methods	AUC (std)	AUPR (std)	Accuracy (std)	F1 (std)
Cold-protein	0.834 (0.028)	0.849 (0.036)	0.747 (0.031)	0.750 (0.039)
Cold-compound	0.986 (0.001)	0.987 (0.001)	0.950 (0.003)	0.955 (0.002)
Blind split	0.845 (0.020)	0.891 (0.022)	0.749 (0.017)	0.779 (0.030)
Random split	0.990 (0.001)	0.990 (0.001)	0.957 (0.002)	0.962 (0.002)

Table S3. Five-fold results of PMF-CPI on DrugAI dataset under different splitting methods

Split methods	AUC (std)	AUPR (std)	Accuracy (std)	F1 (std)
Cold-protein	0.916 (0.015)	0.837 (0.044)	0.868 (0.016)	0.757 (0.048)
Cold-compound	0.934 (0.011)	0.886 (0.035)	0.900 (0.013)	0.814 (0.043)
Blind split	0.846 (0.028)	0.692 (0.076)	0.845 (0.022)	0.649 (0.055)
Random split	0.965 (0.004)	0.939 (0.006)	0.924 (0.003)	0.871 (0.003)

Table S4. Direct test results of pretrained PMF-CPI on selectivity datasets.

Datasets	MSE	Pearson
AR	1.370	0.280
JAK	1.661	-0.057
CYP	0.529	0.218
